# Can Markerless Pose Estimation Algorithms Estimate 3D Mass Centre Positions and Velocities during Linear Sprinting Activities?

**DOI:** 10.3390/s21082889

**Published:** 2021-04-20

**Authors:** Laurie Needham, Murray Evans, Darren P. Cosker, Steffi L. Colyer

**Affiliations:** Centre for the Analysis of Motion, Entertainment Research and Applications, University of Bath, 1 West, Office 5.113, Bath BA2 7AY, UK; me475@bath.ac.uk (M.E.); dpc22@bath.ac.uk (D.P.C.); sc356@bath.ac.uk (S.L.C.)

**Keywords:** OpenPose, computer vision, 3D reconstruction, Kalman smoothing

## Abstract

The ability to accurately and non-invasively measure 3D mass centre positions and their derivatives can provide rich insight into the physical demands of sports training and competition. This study examines a method for non-invasively measuring mass centre velocities using markerless human pose estimation and Kalman smoothing. Marker (Qualysis) and markerless (OpenPose) motion capture data were captured synchronously for sprinting and skeleton push starts. Mass centre positions and velocities derived from raw markerless pose estimation data contained large errors for both sprinting and skeleton pushing (mean ± SD = 0.127 ± 0.943 and −0.197 ± 1.549 m·s^−1^, respectively). Signal processing methods such as Kalman smoothing substantially reduced the mean error (±SD) in horizontal mass centre velocities (0.041 ± 0.257 m·s^−1^) during sprinting but the precision remained poor. Applying pose estimation to activities which exhibit unusual body poses (e.g., skeleton pushing) appears to elicit more erroneous results due to poor performance of the pose estimation algorithm. Researchers and practitioners should apply these methods with caution to activities beyond sprinting as pose estimation algorithms may not generalise well to the activity of interest. Retraining the model using activity specific data to produce more specialised networks is therefore recommended.

## 1. Introduction

The accurate measurement and assessment of athletes’ movement profiles during training and competition can provide coaches with insight into the physical demands of competition and permit the monitoring of an athlete’s physical capabilities over both acute and longitudinal time scales [1]. In winter Olympic sports such as skeleton and bobsleigh, sprinting ability and the ability to load the sled with a high velocity has been associated with high performance outcomes [2,3,4,5] and represents information that coaches can use for talent identification and athlete monitoring.

Arguably, the current gold standard in determining the athlete’s centre of mass (CoM) location during sprinting is through the use of marker-based motion capture [6]. Such systems can reconstruct the location of reflective markers with sub-millimetre accuracy [7] and when placed on the body in anatomically meaningful locations can be used to model the CoM locations of the segments (and thereafter the whole body) with a high precision [8]. However, such methods are often limited to laboratory environments with small capture volumes and highly controlled lighting conditions. Furthermore, the placement of markers is time consuming and may alter technique [9]. To address these problems and allow for field-based collection of CoM displacement and velocity information, a range of technologies have emerged including manually annotated video analysis [10,11], laser distance measurement [12] and global or local positioning systems (GPS and LPS) technology [13].

Computer vision technologies provide an alternative approach to monitoring athlete CoM behaviour during sporting activities. Several semi-automatic, multi-camera vision-based tracking systems are available and in commercial use in team-based sports (e.g., STATS SportVU and Signality). A key advantage of such vision-based systems is that they are non-invasive as the athlete is not required to wear a transceiver. When validated against marker-based motion capture (30 × 30 m outdoor volume) errors in the estimation of total displacement were 2.7% which was comparable to both GPS and LPS errors [6]. Velocity errors were recorded at 0.41 ± 0.08 m⋅s^−1^ and magnitudes of error were found to increase as the speed of the tracking object increased [6]. A possible cause of error in the vision-based systems is the method in which the CoM location is estimated. Vision-based tracking systems typically work by detecting a player and fitting a rectangular bounding box that encompasses the entire player regardless of their body pose [14]. Such an assumption is often violated as performers move through a large range of body configurations during sporting activities. To provide a more accurate representation of the performer’s CoM location it is more appropriate to identify joint centres on the detected person and calculate the CoM location using an inertial model. 

Deep learning-based pose estimation aims to identify body landmarks from regular 2D image data and may provide a robust and non-invasive alternative to bounding-box detection or marker-based motion capture systems to capture information such as CoM position and velocity [9,15]. One such algorithm, OpenPose [16] uses a two-stage convolutional neural network (CNN) to detect a sparse point model of the body. OpenPose is simple to use, provides robust multi-person detection and unlike many pose-estimation models also provides landmarks on the feet and hands which will further assist in the accurate estimation of CoM location. When compared to marker-based motion capture, OpenPose demonstrated 3D joint-centre location errors of between 20 and 40 mm [17] for laboratory-based walking, jumping and throwing. While it is still unknown if such magnitudes of error allow for the accurate calculation of joint kinematics, such an approach may be accurate enough to provide a better estimation of the human mass centre than current vision-based approaches. 

Most CNNs utilise supervised-learning methods [17] using manually annotated points (e.g., joint centres) as training examples. Typically, a model trained via supervised learning methods will generalise well to new data that is similar to the data seen during training. However, researchers using pose-estimation methods in movement sciences should take care to examine the performance of CNN models on the activity in question. To demonstrate this point, in this study we examine two running activities, regular sprinting and skeleton pushing. It is likely that the poses seen during regular sprinting will overlap with those contained within the training data (COCO dataset [18]). However, skeleton pushing provides a more unusual set of poses that may not be well represented in the training data.

Researchers wishing to implement pose estimation algorithms also face further challenges including multi-person tracking, 3D camera fusion before 3D [19] and determining optimum filtering methods for CNN derived data. Indeed, Linke et al. [6] acknowledged that further improvements in vision-based tracking accuracy could be achieved by using more advanced filtering methods. Such tasks are non-trivial, but when implemented correctly may allow for the accurate and reliable reconstruction of 3D keypoints, which in turn may permit the advancement of current athlete CoM location tracking technology. The aim of this study was to evaluate the ability of CNN-based pose-estimation (OpenPose) to estimate CoM velocities during two sprinting activities and examine whether advanced filtering methods can enhance the performance of the vision-based athlete tracking system. 

## 2. Materials and Methods

Twelve international skeleton athletes (seven males (1.81 ± 0.05 m, 83.37 ± 2.73 kg), five females (1.71 ± 0.03 m, 70.04 ± 1.44 kg)) provided written informed consent. Each athlete attended two testing sessions, one at the University of Bath’s outdoor push track and a second at the University of Bath’s indoor sprints track. During the push track session, each athlete performed three maximal effort push starts and during the sprints track session each athlete performed six sprints. 

During both pushing and sprinting trials, motion data were captured concurrently using marker-based and markerless motion capture systems. A 15-camera marker-based motion capture system (Oqus, Qualysis AB, Gothenburg, Sweden) was used to acquire gold-standard data whilst a bespoke 9-camera computer vision system (6 mm lens, 1920 × 1080-pixel resolution, ~90° field of view, JAI sp5000c, JAI ltd, Denmark) captured additional video data. At the push track, both camera systems were positioned around the track in order to capture the pushing action between 5 m and 15 m from the starting block. At the sprints track, both camera systems were positioned around the centre running lane in order to capture the sprinting technique between 0 and 10 m, 10 and 20 m, and 20 and 30 m. 

Motion capture systems were time-synchronised by the custom system’s master frame grabber to achieve a frame locked sampling frequency of 200 Hz in both systems. The Qualisys system was calibrated as per the manufacture’s specifications. The custom camera system was calibrated using a sparse bundle adjustment algorithm to compute camera extrinsic parameters [20] while camera intrinsic parameters were computed according to the method described by [21]. Each motion capture system’s Euclidean space was aligned by moving a single marker randomly through the capture volume. This marker data provided points with which the spatial alignment could be optimised in a least-squares sense. Validity of camera calibration scale was assessed for both camera systems by moving two markers of known distance apart through the capture volume. Markers were tracked by both camera systems and the Euclidean distance between them was computed and compared to the known distance of 601.4 mm.

Forty-four individual markers and four clusters were attached to each participant to create a full body six degrees of freedom (6DoF) model (bilateral feet, shanks and thighs, pelvis and thorax, and bilateral upper arms lower arms, and hands). Following labelling and gap filling of trajectories (Qualisys Track Manager v2019.3, Qualisys, Gothenburg, Sweden) raw trajectories were low-pass filtered using a Butterworth 4th order filter with a cut-off frequency of 12 Hz. Optimal cut-off frequencies were determined by exploiting the properties of the autocorrelation function of white noise [22]. Finally, a 6DoF inverse kinematics (IK) constrained model was computed using Visual 3D (v6, C-Motion Inc., Germantown, MD, USA). Athlete CoM locations were computed [23] for both pushing and sprinting activities and vertical and horizontal CoM velocities were computed using a central finite differences method. Validity of the marker-based CoM model was assessed by fitting a second-order polynomial to the flight phase vertical trajectory data during upright sprinting. The second derivative of each curve was computed via a finite differences method and the result compared against the expected −9.81 m·s^−2^ [24].

**Figure A1:**
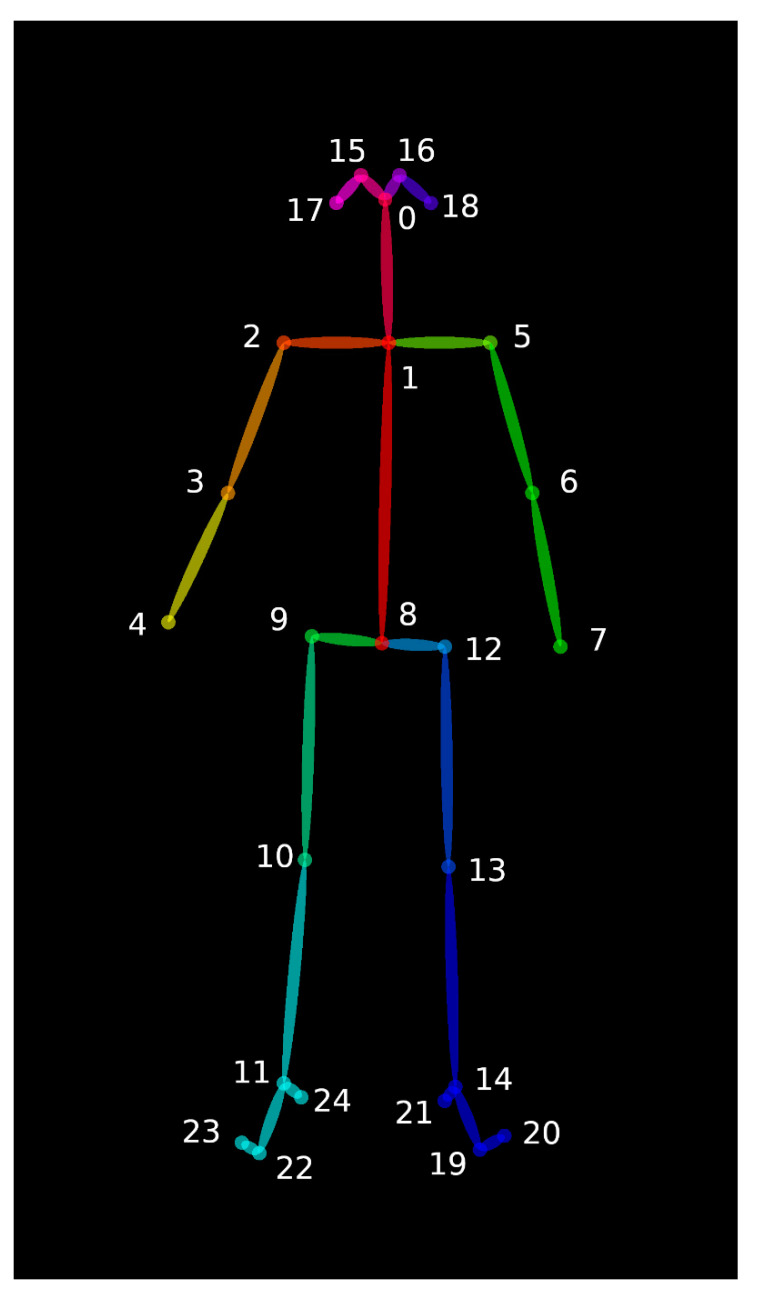
OpenPose Body_25 keypoint model. Adapted from OpenPose GitHub repository [https://github.com/CMU-Perceptual-Computing-Lab/openpose/blob/master/doc/output.md#pose-output-format, accessed on 15 July 2020].

For each field of view, 2D joint centre locations were computed using the OpenPose body_25 model [16] (Table A1, Figure A1) for each detected person in a given camera view. However, OpenPose is only able to provide 2D image planar coordinates and does note associate or ‘track’ each detected perform from frame to frame. As such, an approach based on occupancy maps was used to associate person detections between viewpoints [25]. Firstly, all the 2D feature detections of each person were reduced to a single median point $p_{i,\;v}$ (person i in view v). The observed volume of the track was divided into vertical columns as a grid. Next, each column is projected into each camera view (project the eight corners of the rectangular column and deduce the smallest axis-aligned bounding box that envelopes the projections). For each column k, a count is produced of how many camera views the column is visible in$\;v_{k}$, and for how many views the projected column contains the median point of a person $m_{k}$. Each column is given an occupancy score $o_{k}=\frac{m_{k}}{v_{k}}$. Columns with larger occupancy scores have a higher probability of being at the location of a real person and columns with low visibility can be excluded. Treating the grid of occupancy scores as an image allows for easy identification of peaks in the occupancy map representing individual people. By recording which median points contributed to each column’s scores, per-camera observations are neatly grouped across cameras simply by identifying columns with peak occupancy. Reconstructing a person in 3D can then be achieved by reconstructing each feature in 3D. For example, consider an elbow joint centre observed from the set of 2D detections D. Each detection $d_{i,\;v}\;\in D$ is back projected using the calibration [20,21] of the relevant camera to produce a ray in space $r_{v}=P_{v}\left(d_{i,v}\right)$ (let $P_{v}\left(x\right)$ be a function computing inverse projection and accounting for non-linearities such as lens distortions). A least squares solution can solve for the “intersection” of the 3D rays (closest 3D point to each ray) [26]. However, to account for errors (e.g., mislabelling by the detector or mis-grouping caused by relative position of persons and cameras) a RANSAC [27] process is used to determine the set of inlier rays by using pairs of rays to propose solutions and finding the proposal with which the largest set of rays is consistent. The final 3D position of the feature is taken as the “intersection” of the inliers.

To assess the effects of signal filtering on 3D-fused OpenPose joint centre trajectories, the 3D joint centre coordinates were filtered using two methods. Firstly, a low-pass filter (Butterworth 4th order, cut-off 12 Hz) was implemented as this method is commonly used on marker-based motion capture data. Again, optimal cut-offs were determined using the method described by [22]. Secondly, an optimal fixed-interval Kalman smoother [28] was implemented. The Kalman smoother performs a bi-directional pass to determine an optimal state estimation of a given key point trajectory. Hyperparameters including measurement noise and transition noise were optimised using a grid-search and cross-validation. Finally, using raw, low-pass filtered and Kalman smoothed data, athlete CoM locations were computed for both pushing and sprinting activities using the model described by de Leva [23]. Vertical and horizontal CoM velocities were computed using a central finite differences method.

Agreement between marker and markerless methods was assessed via Bland–Altman analysis and linear regression models. Bland–Altman analysis permits the delineation of systematic (bias) and random (standard deviation of bias) differences between measures with 95% limits of agreement (LoA) [29]. Additionally, we computed linear regression models which provide reliable and sensitive means to compare between biomechanical waveforms [30]. 

## 3. Results

**Figure 1:**
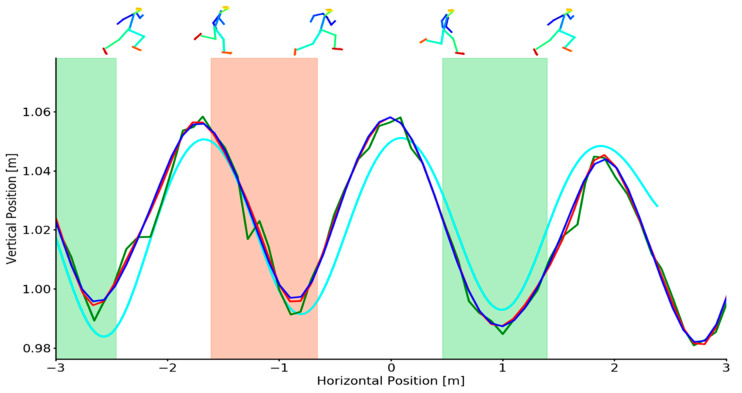
Example individual trial demonstrating sagittal plane CoM positions for criterion (cyan), unfiltered (green), low-pass filtered (red) and Kalman smoothed (blue) data during sprinting. Shaded areas depict the left foot (red shading) and right foot (green shading) stance phase. Footfall events were computed from marker-based foot kinematics [31]. OpenPose joint centre reconstructions (top) at touch-down and toe-off events are overlaid for context.

**Figure 2:**
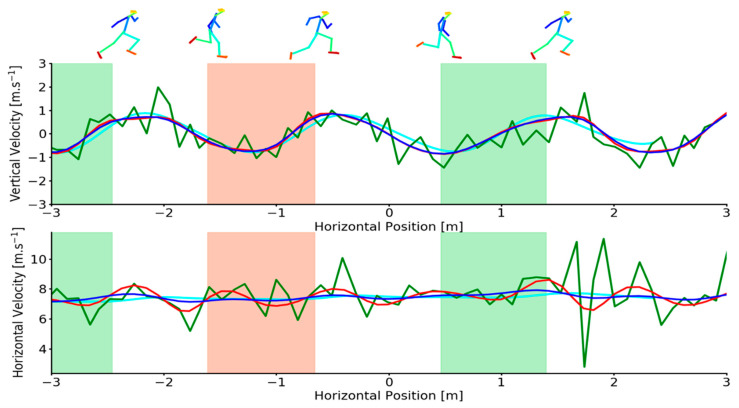
Upper-Example individual trial demonstrating vertical CoM velocities for criterion (cyan), unfiltered (green), low-pass filtered (red) and Kalman smoothed (blue) data as a function of horizontal CoM position during sprinting. Lower -Example individual trial demonstrating horizontal CoM velocities for criterion (cyan), unfiltered (green), low-pass filtered (red) and Kalman smoothed (blue) data as a function of horizontal CoM position during sprinting. Shaded areas depict the left foot (red shading) and right foot (green shading) stance phase. Footfall events were computed from marker-based foot kinematics [31]. OpenPose joint centre reconstructions (top) at touch-down and toe-off events are overlaid for context.

**Figure A2:**
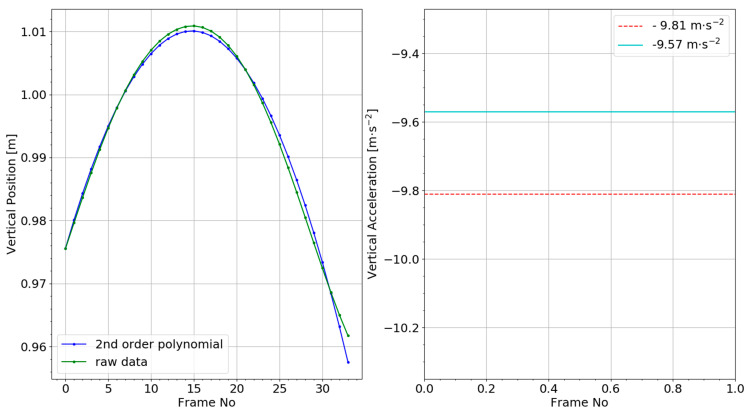
Example CoM model accuracy check for an individual participant’s flight phase using marker-based data. Unfiltered vertical CoM trajectory (left – green) is shown with a 2nd order polynomial fit to the data (left- blue). The double differentiated result (right - cyan) provides an indication of the accuracy of the CoM model.

**Figure A3:**
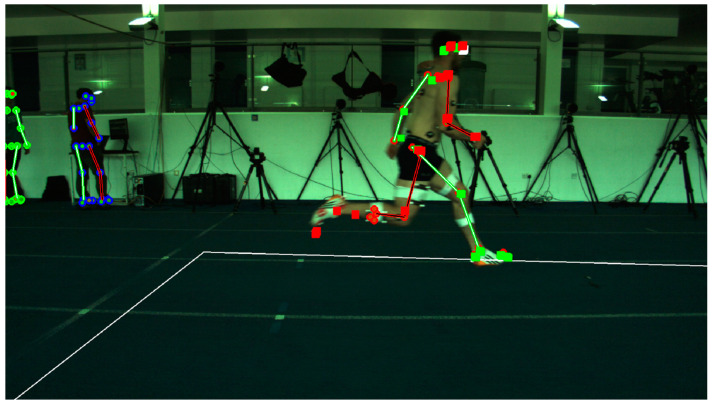
OpenPose keypoint detection examples depicting pose estimation during sprinting. Blue circles depict the 2D OpenPose keypoint locations with the circle fill colour representing the left (green) and right (red) sides of the body. Cubes depict the 3D reconstructed joint centres for the left (green) and right (red) sides of the body projected onto the 2D image plane. This example demonstrates overall good quality 2D detections and 3D reconstruction. Errors in the detection of the right foot can be observed, however, this will largely be corrected during the 3D fusion process which will use detections from other fields of view.

Mean calibration scale accuracy was 0.91 ± 0.76 mm for the marker-based system and 0.74 ± 0.68 mm for the markerless system. Mean CoM vertical acceleration during flight was −9.87 ± 0.42 m·s^−2^ (example provided in Figure A2). Representative examples of OpenPose derived and marker derived CoM trajectories in the sagittal plane during sprinting are shown in Figure 1. Additionally, OpenPose derived CoM position under various filtering conditions are also provided. Representative vertical and horizontal CoM velocities during sprinting are shown in Figure 2. Additional examples demonstrating high and low quality OpenPose joint centres detections during sprinting are provided in the Appendix A (Figure A3). 

**Figure 3:**
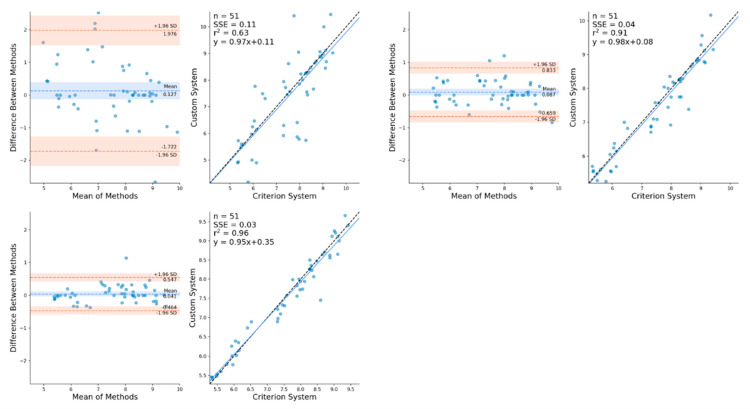
Bland–Altman and linear regression plots demonstrating the mean differences between OpenPose unfiltered (**top-left**), low-pass filtered (**top-right**) and Kalman smoothed (**lower-left**) CoM horizontal velocity against marker-based CoM horizontal velocity during sprinting.

Mean differences (bias) and the SD between OpenPose derived CoM locations and marker derived CoM locations during sprinting remained comparable between unfiltered, low-pass filtered and Kalman smoothed results (Table 1). For the vertical CoM velocity, low-pass filtering and Kalman smoothing demonstrated no improvements in the estimation of CoM vertical velocity (Table 2) but did elicit moderate reductions in SD and modest increases in R^2^. However, large reductions in mean difference, SD and limits of agreement and large improvements in R^2^ were observed for horizontal CoM velocity with the Kalman smoother being most effective at reducing error during sprinting (Table 2, Figure 3). Full Bland–Altman and linear regression results as provided in the Appendix A for sprinting CoM positions (Figure A5 and Figure A6) and sprinting CoM velocities (Figure A9).

**Figure 4:**
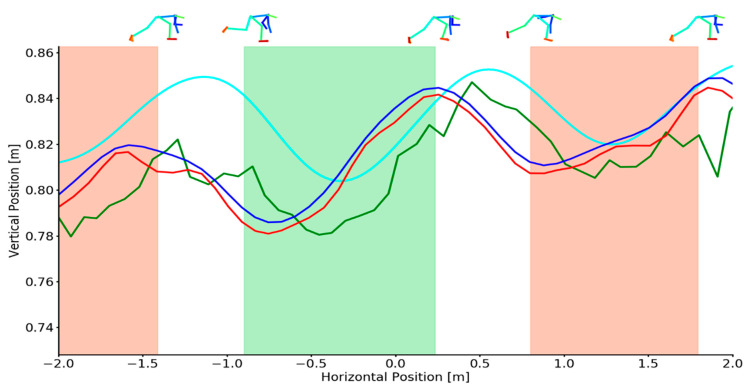
Example individual trial demonstrating sagittal plane CoM positions for criterion (cyan), unfiltered (green), low-pass filtered (red) and Kalman smoothed (blue) data during pushing. Shaded areas depict the left foot (red shading) and right foot (green shading) stance phase. Footfall events were computed from marker-based foot kinematics [31]. OpenPose joint centre reconstructions (top) at touch-down and toe-off events are overlaid for context.

**Figure 5:**
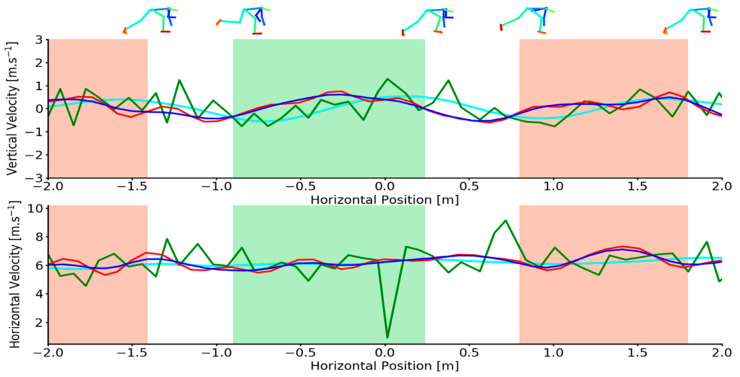
Upper-Example individual trial demonstrating vertical CoM velocities for criterion (cyan), unfiltered (green), low-pass filtered (red) and Kalman smoothed (blue) data as a function of horizontal CoM position during sprinting. Lower-Example individual trial demonstrating horizontal CoM velocities for criterion (cyan), unfiltered (green), low-pass filtered (red) and Kalman smoothed (blue) data as a function of horizontal CoM position during pushing. Shaded areas depict the left foot (red shading) and right foot (green shading) stance phase. Footfall events were computed from marker-based foot kinematics [31]. OpenPose joint centre reconstructions (top) at touch-down and toe-off events are overlaid for context.

**Figure A4:**
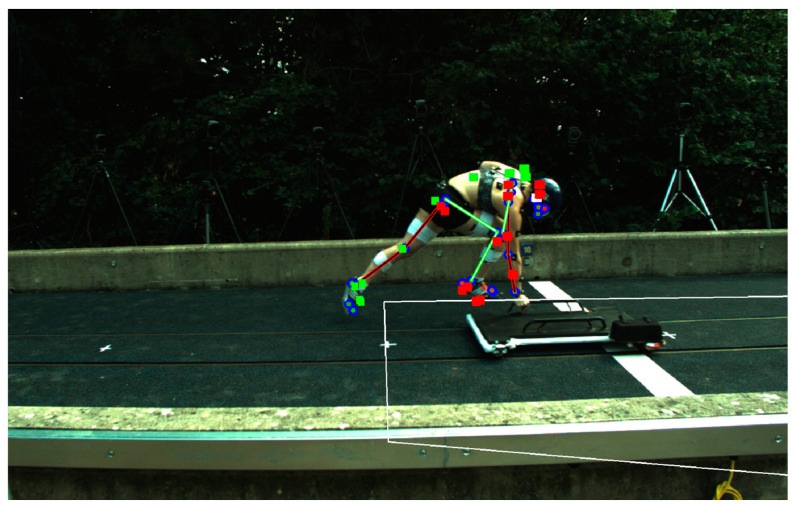
OpenPose keypoint detection examples depicting pose estimation during pushing. Blue circles depict the 2D OpenPose keypoint locations with the circle fill colour representing the left (green) and right (red) sides of the body. Cubes depict the 3D reconstructed joint centres for the left (green) and right (red) sides of the body projected onto the 2D image plane. This example demonstrates that limb switching has occurred for the legs but this has largely been corrected during the 3D fusion process. However, further issues can been seen with the left arm where OpenPose has detected joint centres with limited success in multiple fields of view.

**Figure A5:**
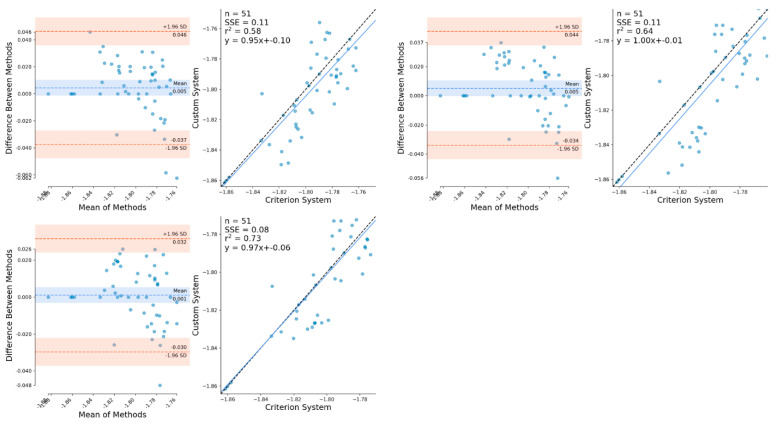
Bland-Altman and linear regression plots demonstrating the differences between OpenPose unfiltered (top-left), low-pass filtered (top-right) and Kalman smoothed (lower-left) CoM horizontal position against marker-based CoM horizontal position during sprinting.

**Figure A6:**
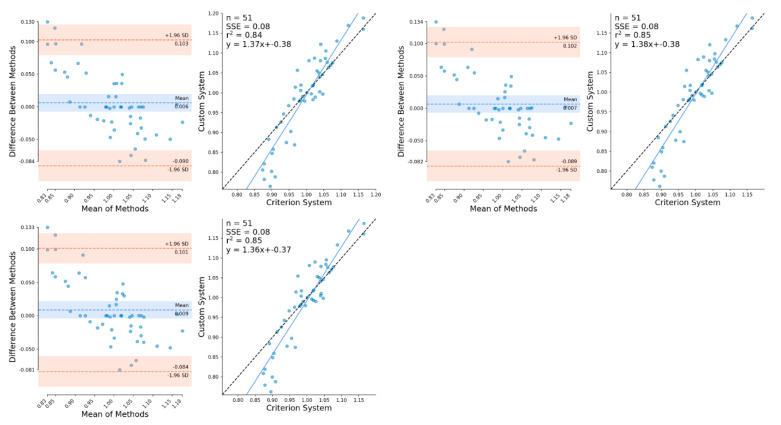
Bland-Altman and linear regression plots demonstrating the differences between OpenPose unfiltered (top-left), low-pass filtered (top-right) and Kalman smoothed (lower-left) CoM vertical position against marker-based CoM vertical position during sprinting.

Representative examples of OpenPose derived and marker derived CoM trajectories in the sagittal plane during skeleton pushing are shown in Figure 4. Additionally, OpenPose derived CoM position under various filtering conditions are also provided. Representative vertical and horizontal CoM velocities during sprinting are shown in Figure 5. Additional examples demonstrating high and low quality OpenPose joint centres detections during pushing are provided in the Appendix A (Figure A4).

**Figure A7:**
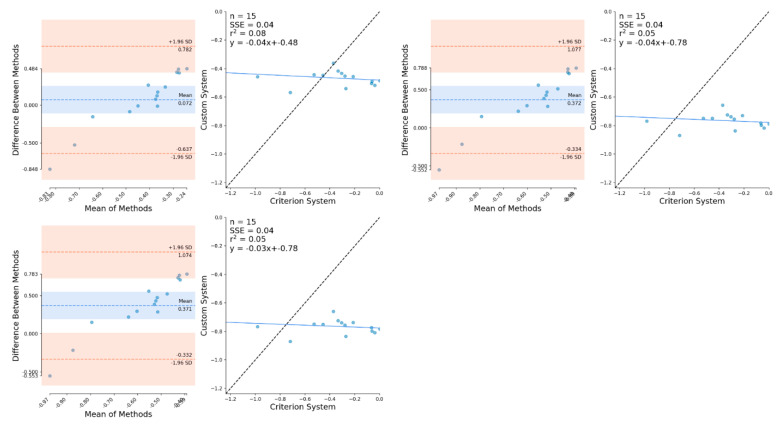
Bland-Altman and linear regression plots demonstrating the differences between OpenPose unfiltered (top-left), low-pass filtered (top-right) and Kalman smoothed (lower-left) CoM horizontal position against marker-based CoM horizontal position during pushing.

**Figure A8:**
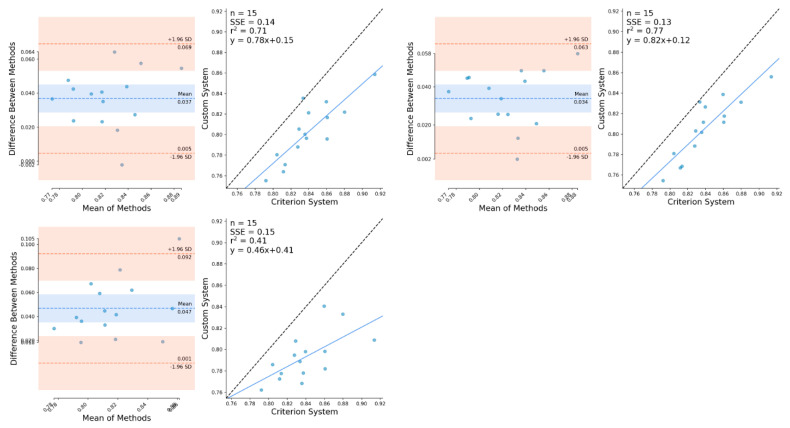
Bland-Altman and linear regression plots demonstrating the differences between OpenPose unfiltered (top-left), low-pass filtered (top-right) and Kalman smoothed (lower-left) CoM vertical position against marker-based CoM vertical position during pushing.

**Figure A9:**
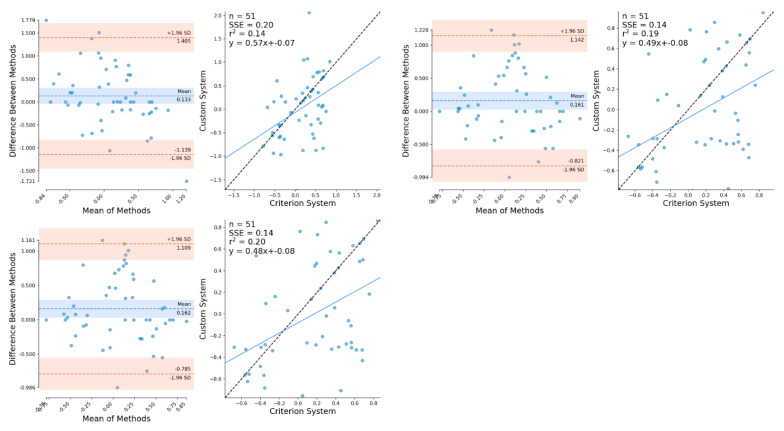
Bland-Altman and linear regression plots demonstrating the differences between OpenPose unfiltered (top-left), low-pass filtered (top-right) and Kalman smoothed (lower-left) CoM vertical velocity against marker-based CoM vertical velocity during sprinting.

**Figure A10:**
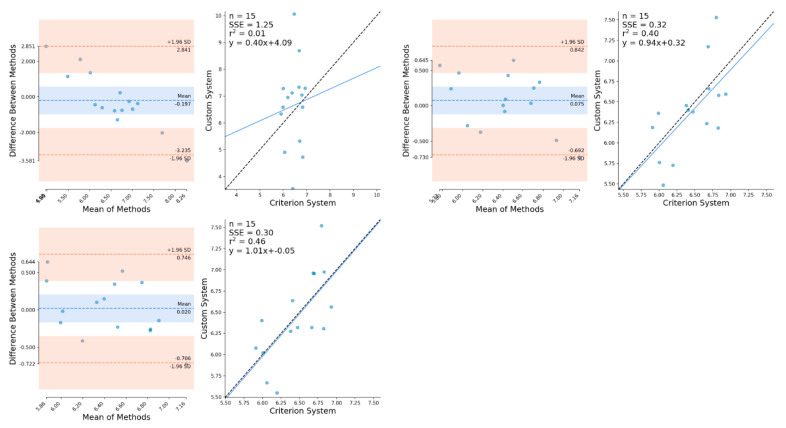
Bland-Altman and linear regression plots demonstrating the differences between OpenPose unfiltered (top-left), low-pass filtered (top-right) and Kalman smoothed (lower-left) CoM horizontal velocity against marker-based CoM horizontal velocity during pushing.

**Figure A11:**
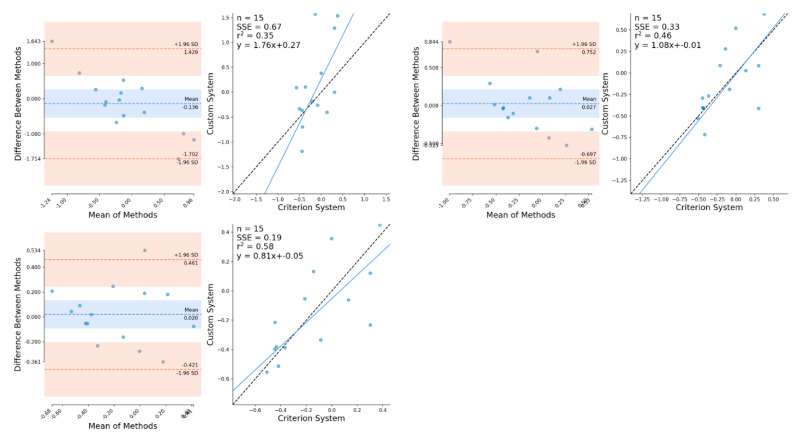
Bland-Altman and linear regression plots demonstrating the differences between OpenPose unfiltered (top-left), low-pass filtered (top-right) and Kalman smoothed (lower-left) CoM vertical velocity against marker-based CoM vertical velocity during pushing.

Mean differences (bias) between OpenPose derived CoM locations and marker derived CoM locations during pushing remained comparable between unfiltered, low-pass filtered and Kalman smoothed results (Table 3) although filtering substantially improved the SD and limits of agreement for horizontal position. Both horizontal and vertical CoM velocity differences were high for unfiltered OpenPose data but greatly improved in performance under low-pass filtering and Kalman smoothing (Table 4). Full Bland–Altman and linear regression results as provided in the Appendix for pushing CoM positions (Figure A7 and Figure A8) and sprinting CoM velocities (Figure A10 and Figure A11).

## 4. Discussion

The non-invasive, accurate and reliable measurement of CoM behaviour provides a rich data source for coaches assessing a range of movement characteristics. In this study, CNN-based pose estimation (OpenPose) and advanced signal processing techniques were used to evaluate a non-invasive method capable of capturing CoM behaviour. 

For the sprinting activity, OpenPose derived CoM positions were generally predicted to within 5 cm of the criterion CoM position but with a tendency to systematically over and underestimate the vertical oscillations of the CoM during the gait cycle (Figure 1). Furthermore, horizontal and vertical CoM velocities derived from unfiltered OpenPose joint centre trajectories exhibited large mean errors and standard deviations (0.127 ± 0.943 m·s^−1^ and 0.133 ± 1.405 m·s^−1^—Table 2) when compared to data derived from marker-based motion capture. Both the motion capture systems demonstrated high reconstruction accuracy (mean < 1 mm) and acceptable marker derived CoM accuracy. As such markerless CoM errors were primarily attributed to the 2D pose estimator where joint centre errors of >40 mm have been reported [17] which could indeed account for the CoM position differences that were observed in this study. Moreover, high frequency keypoint jitter, which often arises when a joint centre is predicted with low confidence and as such the predicted location jumps around by a small amount between frames likely further contributed to position and velocity errors (examples in Appendix A Figure A3). The misidentification of a joint centre or erroneous contra-lateral switching of entire limbs was also observed during data processing and as such may have contributed artefact to the CoM position and velocity estimation. In this study we attempted to attenuate such errors by fusing the 2D points early in our 3D reconstruction pipeline in order to allow for the identification and correction of erroneous joint centre detections and limb switching. Nonetheless, the large magnitudes of error in CoM velocity and pose estimation associated artefacts demonstrate that researchers and practitioners should use unfiltered OpenPose data with caution. 

Very large magnitudes of error were observed for horizontal and vertical CoM position and velocity during the skeleton pushing activity (−0.197 ± 1.549 m·s^−1^ and −0.136 ± 0.798 m·s^−1^—Table 4) for unfiltered OpenPose data and align with the findings of previous work where large errors were reported for step averaged values [32]. In addition to the sources of error highlighted for the sprinting activity, during the pushing activity, OpenPose was often unable to correctly identify the majority of joint centre locations (examples in Appendix A Figure A4). As such the 3D reconstructed joint centres did not accurately represent the skeleton athlete’s joint configurations leading to extremely large errors in CoM position and velocity estimations. CNN-based pose estimation algorithms such as OpenPose are trained on tens of thousands of labelled images [18]. However, a known limitation of such supervised deep learning methods is their ability to generalise beyond the poses that are represented in the training data set [33]. While the poses exhibited during sprinting may have been reasonably well represented during the training of OpenPose, it appears that the unusual body poses exhibited during skeleton pushing were not as well represented during training and as such OpenPose struggled to correctly detect joint centre locations in these image sequences. This point is also demonstrated by Seethapathi et al. [34] where pose estimation algorithms perform poorly when applied to gymnastics sequences. This raises an important consideration for researchers and practitioners wishing to apply pose estimation methods to sporting activities that do not feature in the training data. 

The presence of artefact in a signal is commonplace in many biological waveforms. As such many methods have emerged that permit the smoothing of raw signals with the aim of reducing the presence of noise. The magnitudes of error observed in the unfiltered OpenPose data suggests that the use of signal processing techniques is required to reduce the presence of signal artefact, not unlike data recorded using marker-based motion capture. Filtering the position data did little to improve overall CoM location performance in either activity but had a substantial effect on the calculation of CoM velocity where the differentiation process amplified error in the signal. For example, a further consequence of high frequency keypoint jitter was observed in the small but sudden changes it caused to CoM position. When the CoM position was differentiated these small but high frequency artefacts were amplified and presented as large errors in CoM velocity. In this study we addressed this problem with signal processing techniques, however, an alternative approach could be to implement pose estimation methods that use image sequences rather than just a single image to predict keypoint locations and as such can learn both spatial and temporal information about keypoint locations. Such methods (e.g., Pose Flow [35], Spatio-Temporal Affinity Fields [36]) may be able to produce a more temporally smooth signal that is more robust to errors caused by keypoint jitter and switching that were encountered using OpenPose in this study. Indeed, several studies using OpenPose data have reported heavily filtering the joint centre trajectories using either a Butterworth low-pass filter (cut-off frequency-2 Hz [17]) or a weighted moving average filter [37]. Butterworth low-pass filters are widely used by biomechanics researchers [38] and demonstrated the ability to reduce the magnitudes of error in the OpenPose data in this study (Table 2 and Table 4, Figure 2). However, the Butterworth low-pass filter did demonstrate sensitivities to large outliers for example, Figure 2 (lower) between approximately 1.5 and 2 m on the *x*-axis. In contrast, Kalman smoothing proved more robust to large outliers and in most cases reduced measurement errors most effectively (Table 2 and Table 4). While rarely used in biomechanics research, Kalman filtering methods are popular in computer vision applications for object tracking tasks [39]. When treated with a Kalman smoother, OpenPose derived CoM horizontal velocity demonstrated comparable accuracy to other non-invasive field-based measures of sprinting performance. For the measurement of horizontal CoM velocity, laser distance measurement has reported errors of up to 0.41 m·s^−1^ [12], semi-automatic multiple-camera video technology has reported errors of 0.41 ± 0.18 m·s^−1^ [6] and GPS errors of 0.28 ± 0.07 m·s^−1^ [6] when compared to errors of −0.041 ± 0.257 m·s^−1^ (Table 2) from this study. However, researchers using pose estimation methods such as OpenPose are encouraged to err on the side of caution as in practice the large limits of agreement (low precision) reported here reduce the ability of such methods to detect small and potentially meaningful changes in sprinting and pushing performance.

Using OpenPose has the potential to provide reasonable estimations of CoM position and velocity during sprinting. There is however a limit to the denoising capabilities of Butterworth low-pass filters or Kalman smoothers, for example, during pushing (Figure 4 and Figure 5). The majority of measurement errors that were observed in this study are assumed to originate from the inaccurate or erroneous detection of joint centres by OpenPose. Seethapathi et al. [34] highlights that pose estimation methods present enormous potential for movement sciences such as biomechanics but also acknowledge that better pose estimation algorithms are required for such applications. Further study is required to better understand the accuracy of joint centre detection by pose estimation algorithms but in the meantime, there are several steps that future work should consider. For sports such as skeleton where unusual body poses may fall beyond the generalisability of current pose estimation algorithms, networks can be retrained and specialised via transfer learning [40,41] to improve performance on a specific task. Open-source libraries such as DeepLabCut [42] provide an effective way to achieve this with the potential to improve joint centre estimation performance. However, more broadly, for pose estimation methods to become more effective, there is a need for large, open-access, high quality biomechanics data sets [34] which can be used to train the next generation of pose estimation algorithms. 

Despite the limitations presented by pose-estimation algorithms in this study, pose estimation is a rapidly advancing field that provides huge potential to capture CoM behaviour in a real-world environment and in a non-invasive manner. The approach presented in this study is capable of integrating the latest pose estimation models to enhance CoM location estimation and could be scaled up to capture data in larger volumes with multiple athletes. In the near future such approaches will likely outperform currently used technologies such as laser distance measurement and vision-based local positioning systems.

## 5. Conclusions

In this study we demonstrate that OpenPose derived CoM positions and velocities have the potential to provide an accurate non-invasive alternative to current technologies. However, limitations in precision may reduce the ability to detect small and potentially meaningful changes in sprinting and pushing performance. This approach was compared against marker-based motion capture and demonstrated improved performance when data were treated with a Kalman smoother. However, large errors due to poor joint centre localisation were observed, especially for unfiltered OpenPose data, and during less common sporting activities such as skeleton push starts. The method presented in this study has the potential to provide valid estimations of CoM velocities during sprinting, but researchers and practitioners should be careful when applying pose estimation methods to sports that contain less common body poses.

## Figures and Tables

**Table 1:** Between system comparison of CoM position during sprinting.

CoM Position	Component	Mean Difference (Bias) (m)	±SD	95% LoA	R^2^
Unfiltered	Horizontal	0.005	0.021	−0.037–0.046	0.58
Unfiltered	Vertical	0.006	0.028	−0.090–0.103	0.84
Low-Pass	Horizontal	0.005	0.020	−0.034–0.044	0.64
Low-Pass	Vertical	0.007	0.027	−0.089–0.102	0.85
Kalman	Horizontal	0.001	0.016	−0.030–0.032	0.73
Kalman	Vertical	0.009	0.032	−0.084–0.101	0.85

**Table 2:** Between system comparison of CoM velocity during sprinting.

CoM Velocity	Component	Mean Difference (Bias) (m·s^−1^)	±SD	95% LoA	R^2^
Unfiltered	Horizontal	0.127	0.943	−1.722–1.974	0.63
Unfiltered	Vertical	0.133	0.648	−1.139–1.405	0.14
Low-Pass	Horizontal	0.087	0.381	−0.659–0.833	0.91
Low-Pass	Vertical	0.161	0.501	−0.821–1.142	0.19
Kalman	Horizontal	0.041	0.257	−0.464–0.547	0.96
Kalman	Vertical	0.162	0.483	−0.785–1.109	0.20

**Table 3:** Between system comparison of CoM position during pushing.

CoM Position	Component	Mean Difference (Bias) (m)	±SD	95% LoA	R^2^
Unfiltered	Horizontal	0.072	1.549	−0.637–0.786	0.08
Unfiltered	Vertical	0.037	0.016	0.005–0.069	0.71
Low-Pass	Horizontal	0.372	0.391	−0.334–1.077	0.05
Low-Pass	Vertical	0.034	0.014	0.005–0.063	0.77
Kalman	Horizontal	0.371	0.370	−0.332–1.074	0.05
Kalman	Vertical	0.047	0.023	0.001–0.092	0.41

**Table 4:** Between system comparison of CoM velocity during pushing.

CoM Velocity	Component	Mean Difference (Bias) (m·s^−1^)	±SD	95% LoA	R^2^
Unfiltered	Horizontal	−0.197	1.549	−3.235–2.841	0.01
Unfiltered	Vertical	−0.136	0.798	−1.702–1.429	0.35
Low-Pass	Horizontal	0.075	0.391	−0.692–0.842	0.40
Low-Pass	Vertical	0.027	0.369	−0.697–0.752	0.46
Kalman	Horizontal	0.020	0.370	−0.706–0.746	0.46
Kalman	Vertical	0.020	0.235	−0.421–0.461	0.58

## Data Availability

Not applicable.

## Appendix A

The OpenPose (Cao et al., 2017) body part mapping order is given in Table A1 and Figure A1. These points are output by the Body_25 model which is derived from the COCO training data set (Lin et al., 2014) and Human Foot Keypoint Dataset (Cao et al., 2017).

**Table A1:** OpenPose Body_25 body part mapping guide.

Keypoint Number	Body Part	Dataset
**0**	Nose	COCO
**1**	Neck	COCO
**2**	Right Shoulder	COCO
**3**	Right Elbow	COCO
**4**	Right Wrist	COCO
**5**	Left Shoulder	COCO
**6**	Left Elbow	COCO
**7**	Left Wrist	COCO
**8**	Mid Hip	COCO
**9**	Right Hip	COCO
**10**	Right Knee	COCO
**11**	Right Ankle	COCO
**12**	Left Hip	COCO
**13**	Left Knee	COCO
**14**	Left Ankle	COCO
**15**	Right Eye	COCO
**16**	Left Eye	COCO
**17**	Right Ear	COCO
**18**	Left Ear	COCO
**19**	Right Big Toe	Human Foot Keypoint Dataset
**20**	Right Small Toe	Human Foot Keypoint Dataset
**21**	Right Heel	Human Foot Keypoint Dataset
**22**	Left Big Toe	Human Foot Keypoint Dataset
**23**	Left Small Toe	Human Foot Keypoint Dataset
**24**	Left Heel	Human Foot Keypoint Dataset
**25**	Background	COCO
